# Prion-like characteristics of the bacterial protein Microcin E492

**DOI:** 10.1038/srep45720

**Published:** 2017-03-31

**Authors:** Mohammad Shahnawaz, Kyung-Won Park, Abhisek Mukherjee, Rodrigo Diaz-Espinoza, Claudio Soto

**Affiliations:** 1 Mitchell Center for Alzheimer’s disease and related Brain Disorders, Department of Neurology, University of Texas Houston Medical School, Houston, Texas, USA

## Abstract

Microcin E492 (Mcc) is a pore-forming bacteriotoxin. Mcc activity is inhibited at the stationary phase by formation of amyloid-like aggregates in the culture. Here we report that, in a similar manner as prions, Mcc naturally exists as two conformers: a β-sheet-rich, protease-resistant, aggregated, inactive form (Mcc^ia^), and a soluble, protease-sensitive, active form (Mcc^a^). The exogenous addition of culture medium containing Mcc^ia^ or purified *in vitro*-generated Mcc^ia^ into the culture induces the rapid and efficient conversion of Mcc^a^ into Mcc^ia^, which is maintained indefinitely after passaging, changing the bacterial phenotype. Mcc^ia^ prion-like activity is conformation-dependent and could be reduced by immunodepleting Mcc^ia^. Interestingly, an internal region of Mcc shares sequence similarity with the central domain of the prion protein, which is key to the formation of mammalian prions. A synthetic peptide spanning this sequence forms amyloid-like fibrils *in vitro* and is capable of inducing the conversion of Mcc^a^ into Mcc^ia^ *in vivo*, suggesting that this region corresponds to the prion domain of Mcc. Our findings suggest that Mcc is the first prokaryotic protein with prion properties which harnesses prion-like transmission to regulate protein function, suggesting that propagation of biological information using a prion-based conformational switch is an evolutionary conserved mechanism.

Prions are the infectious agents responsible for a group of neurodegenerative diseases, termed prion diseases or transmissible spongiform encephalopathies (TSEs), including scrapie, chronic wasting disease, bovine spongiform encephalopathy, and Creutzfeldt-Jakob disease^1^,^2^,^3^. The main component of a prion is a misfolded form of the normal host-encoded prion protein (PrP^C^) capable of self-perpetuating by converting PrP^C^ into the pathological, infectious form (PrP^Sc^). Although the molecular mechanism through which conversion of PrP^C^ to PrP^Sc^ occurs is poorly understood, the experimental evidence suggests a direct interaction of PrP^C^ with pre-existing oligomeric PrP^Sc^, which acts as a seed to induce the recruitment of the normal protein into the aggregate, templating the conformational conversion of PrP^C^ into PrP^Sc^ (ref. 4). The conversion is associated with an increase in β-sheet structure, insolubility in ionic detergents, resistance to proteolytic degradation, and the formation of amyloid-like aggregates^5^,^6^. *In vitro*, prion protein polymerizes through a nucleation-dependent process, which is composed of a lag phase followed by an extension phase^7^,^8^,^9^. The lag phase can be reduced or removed either by increasing protein concentration or by the addition of preformed aggregates. *In vivo,* injection or ingestion of purified PrP^Sc^ or tissue extracts containing PrP^Sc^ from infected animals leads to the pathological conversion of host PrP^C^ into PrP^Sc^ and the development of prion disease. Thus, the seeding/nucleation mechanism offers a plausible model for the infectious nature of prions^8^.

Analogous to mammalian prions, a number of self-perpetuating prions have been identified in the yeast *Saccharomyces cerevisiae*, which are responsible for heritable changes in phenotype^10^,^11^,^12^,^13^. The [*PSI*^+^], [*URE*^+^] and [*RNQ*^+^] are extensively characterized prions of Sup35p, Ure2, and Rnq1 proteins, respectively. The [*PSI*^+^] is an inactive aggregated form of Sup35p, a subunit of a translation termination factor, whereas the [*psi*^−^] is an active soluble form of Sup35p. The Sup35 protein consists of three domains; an N-terminal region (N), which corresponds to the prion-forming domain (PrD) and is required for induction and propagation of the prion form. The C-terminal domain (C) is necessary for translation termination, whereas the highly charged middle domain (M) increases the solubility of the protein. The prion domain of yeast is modular and can confer the ability to form prions when fused to other proteins. The heat-shock protein 104 (Hsp104) plays a key role in yeast prion propagation by fragmenting large polymers, and inhibition of its activity by guanidine hydrochloride cures yeast prions. Unlike mammalian prions, yeast prions are not generally considered pathogenic, although this is still a subject under debate^14^. Rather, they appear to provide a fitness advantage under adverse conditions^8^,^15^,^16^,^17^. Prion-like phenomena have also been shown for several other proteins in diverse organisms. For example, the neuronal isoform of *Aplysia* cytoplasmic polyadenylation element binding protein (CPEB) has been shown to undergo prion-like conformational changes that help to stabilize long term memory^18^. The human mitochondrial antiviral signaling (MAVS) protein has been described to use the prion-like mechanism to activate and propagate antiviral innate immune signals^19^. These reports provide evidence that the prion-like self-replication of conformational changes in a protein is not always pathogenic; rather, such conformational changes may regulate protein function and provide a selectable advantage in extreme conditions.

In this study, we show prion-like, self-propagation of conformational changes in the bacteriotoxin Mcc that modulate the biological activity of the protein. Mcc is a low molecular weight (~7.8 kDa) antibiotic produced by *Klebsiella pneumoniae* RYC492, which is active against many *Enterobacteriaceae* species that compete with *Klebsiella*^20^,^21^,^22^. Mcc kills sensitive bacteria by forming ion-channels on the cell membrane^22^,^23^,^24^. Active Mcc is produced only during the exponential phase of growth^21^,^25^. Loss of activity of Mcc in the stationary phase is neither due to differences in amounts of protein secreted, nor degradation or posttranslational modifications^26^. Rather, the loss of Mcc activity appears to be due to the conversion of soluble protein into amyloid-like fibrils, similar to those associated with many human diseases including Alzheimer’s, Parkinson’s, Diabetes type II, and prion diseases^27^. Mcc aggregates formed *in vitro* or *in vivo* share typical characteristics with disease-associated protein aggregates, including β-sheet rich secondary structure, ultrastructural morphology of the aggregates as long unbranched amyloid fibrils, binding to amyloid specific dyes and partial resistance to proteolysis^27^.

## Results

### Two forms of Mcc are functionally, biochemically, and structurally distinct

**Figure 1:**
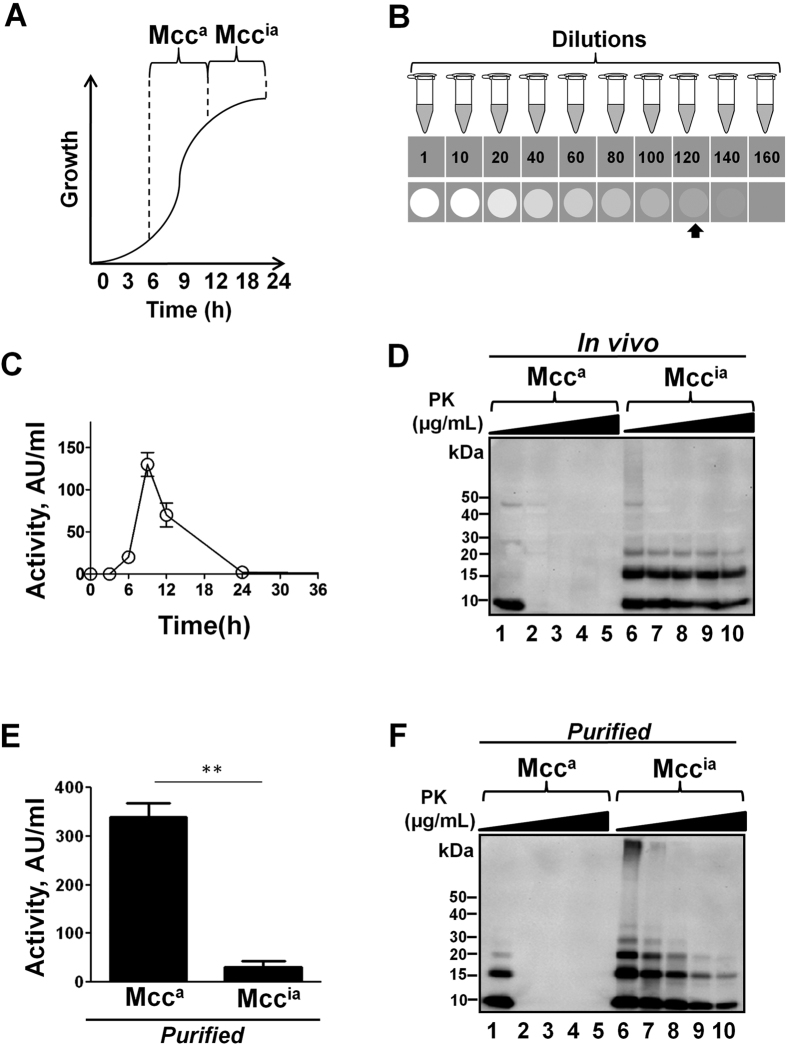
Two functionally and biochemically distinct forms of Mcc. (**A**) A schematic diagram of the natural production of two forms of microcin at different stages of the bacterial growth phase; Mcc^a^ (active form of Mcc) and Mcc^ia^ (inactive form of Mcc). (**B**) A scheme of the critical dilution method (CDM) to measure Mcc bacteriotoxin activity. In brief, samples were centrifuged and supernatants were laid onto LB agar plates overlaid with Mcc-sensitive *E.coli*. After 16 h of incubation at 37 °C, the activity of Mcc in the last dilution that gave detectable inhibition (signal approximately 2-times above the background levels as indicated by an arrow) was determined and expressed in arbitrary units(AU)/ml. **C**: Typical changes on Mcc activity during different stages of culture growth. Mcc producing bacteria were grown in minimal medium at 37 °C. Aliquots were removed periodically, and activity was measured by CDM (see “Materials and Methods”). Activity was measured in duplicate samples and *error bars* indicate standard deviation (S.D). (**D**) Structural changes of Mcc reflected by differences on resistance to proteolytic degradation. Immunoblots of PK-treated aliquots of culture at the exponential and stationary phase, containing Mcc^a^ (9 h in culture) and Mcc^ia^ (24 h in culture), respectively. Samples in *Lanes* 2-5 and 7–10, were treated with increasing concentrations of PK (1, 10, 100 and 1000 μg/ml). Samples in lanes 1 and 6 were left untreated. **E**: The activity of purified Mcc^a^ (400 μg/ml) before and after aggregation into Mcc^ia^ at 37 °C for 24 h was measured by CDM. Error bars indicate S.D. The differences were statistically analyzed by the student t-test (** P = 0.0052). **F**: Acquisition of protease-resistance upon *in vitro* conversion of Mcc^a^ into Mcc^ia^. Aliquots of purified Mcc^a^ (t = 0 h) and after conversion into Mcc^ia^ (t = 24 h) were subjected to the same treatment with PK as described in *panel* D. Mcc^a^ (*lanes* 1-5) and Mcc^ia^ (*lanes* 6-10). Samples in *Lanes* 2-5 and 7-10, were treated with increasing concentrations of PK (1, 10, 100 and 1000 μg/ml). Samples in lanes 1 and 6 were left untreated.

Two distinct forms of Mcc have been observed during the growth phase of *Klebsiella pneumoniae* RYC492 strain^21^,^25^. The active form of Mcc (hereafter designated as Mcc^a^) is produced during the exponential phase of growth, while the inactive form of Mcc (hereafter termed Mcc^ia^) appears during the stationary phase (Fig. 1A). Such a phenomenon could easily be replicated by growing Mcc producing *E. coli* VCS257 cells harboring the pJEM15 plasmid in M9 minimal medium at 37 °C. Mcc activity was determined by the critical dilution method (CDM), as described in “Materials and Methods” (Fig. 1B). The activity of Mcc increased exponentially, peaking at 9 h in the exponential phase. The activity started to decline around 12 h, becoming undetectable at 24 h in the stationary phase (Fig. 1C). The loss of Mcc activity in the stationary phase was paralleled by the formation of an altered conformation of Mcc that resulted in an increase in protease resistance. For this experiment, aliquots of culture medium from stationary and exponential phases were subjected to limited proteolysis with proteinase K (PK; 1–1000 μg/ml) for 30 min at 37 °C. The aliquots coming from the stationary phase, containing mostly Mcc^ia^, showed a remarkable resistance to proteolysis compared to Mcc^a^, coming from the exponential phase, as shown by immunoblot analysis (Fig. 1D). Strikingly, a large proportion of Mcc^ia^ remained undigested even after incubation with a very high concentration of PK (1000 μg/ml), resembling the behavior of PrP^Sc^.

The conversion of Mcc^a^ into Mcc^ia^ that occurs *in vivo* during the bacteria culture growth was reproduced *in vitro* using purified Mcc. The highly purified Mcc^a^ (400 μg/ml) from the exponential phase (9 h) of Mcc producing bacteria was allowed to aggregate in 50 mM PIPES, pH 6.5 containing 500 mM NaCl at 37 °C and the activity of Mcc before (0 h) and after 24 h of incubation was determined by CDM. Purified Mcc^a^ before aggregation was highly active, whereas after 24 h of incubation, Mcc activity diminished substantially (P = 0.0052), resembling the *in vivo* conversion of Mcc^a^ into Mcc^ia^ (Fig. 1E). The change in bacteriotoxin activity by *in vitro* aggregation was associated with the acquisition of protease resistance. An immunoblot analysis before and after PK digestion clearly showed that Mcc^a^ was sensitive to PK, whereas *in vitro*-generated Mcc^ia^ (by 24 h of incubation) exhibited remarkable resistance to PK (Fig. 1F). These findings indicate that Mcc structural changes, in the absence of any other factor, are responsible for the functional changes in biological activity.

### Mcc^ia^ is a β-sheet rich, amyloid-like aggregate

**Figure 2:**
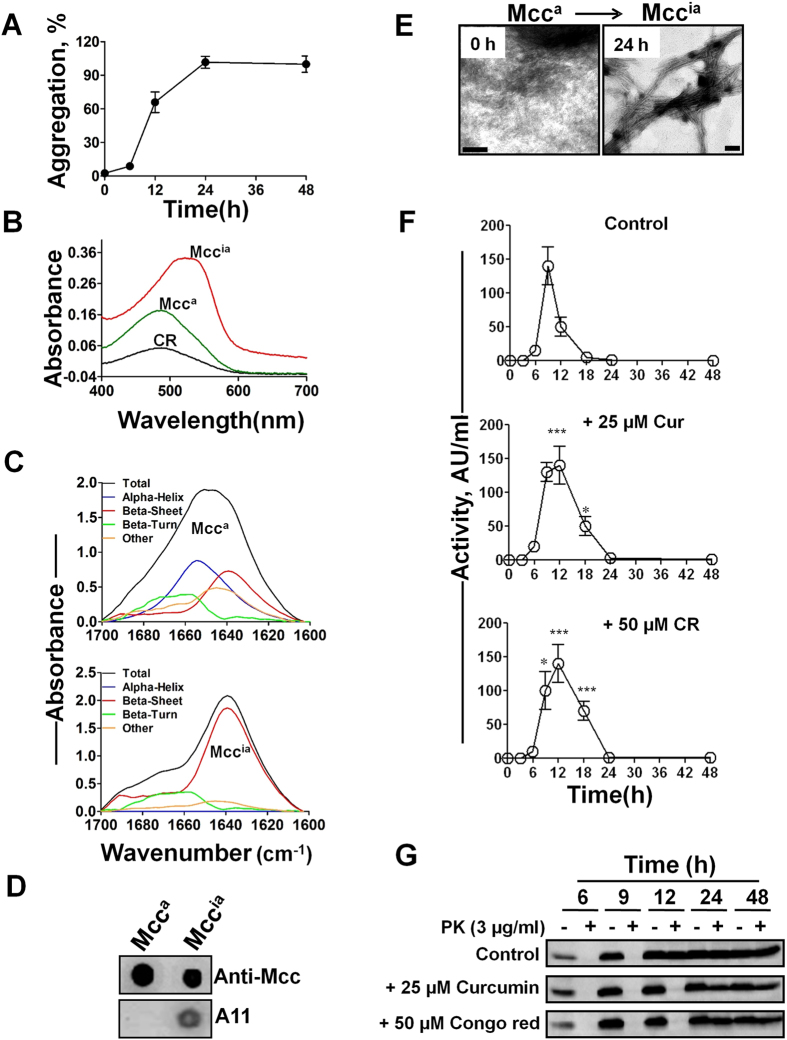
Mcc^ia^ is a β-sheet rich amyloid aggregate. (**A**) Purified Mcc^a^ (400 μg/ml) was allowed to aggregate at 37 °C for 48 h and the extent of amyloid formation was monitored by the ThT binding assay. Experiment was done in duplicate and data correspond to the average ± S.D. (**B**) Absorption spectra of bound CR in the presence of purified Mcc^a^ (*green line*) and Mcc^ia^ (*red line*) produced after aggregation of Mcc^a^ for 24 h at 37 °C. Black line represents CR alone. (**C**) FTIR spectra of purified Mcc^a^ (*top panel*) and Mcc^ia^ (*bottom panel*). The black line represents the experimental spectra and the colored lines are the deconvoluted components corresponding to the various structural motifs as obtained by computer analysis. The peak assignments was as following: ~1639 and ~1691 cm^−1^ for β sheets; ~1654 cm^−1^ for α-helix; ~1648 cm^−1^ for random structure; ~1667,~1675 and ~1680 cm^−1^ for β turns^29^. (**D**) Dot blot analysis for the interaction of Mcc^a^ and Mcc^ia^ with the A11 conformational antibody that recognizes β-sheet-rich soluble oligomers. (**E**) Ultrastructural morphologies of Mcc^a^ and Mcc^ia^ under TEM after negative staining. The bars represent 100 nm. (**F**) Known amyloid inhibitors delay conversion of Mcc^a^ into Mcc^ia^. Mcc producing bacteria were grown in minimal medium at 37 °C until 48 h either in the absence (*control*), or in the presence of Curcumin (25 μM) and CR (50 μM). Aliquots were removed periodically and activity of Mcc was measured by the critical dilution method. Activity was measured in duplicate samples and *error bars* indicate S.D. The differences in Mcc activity between control and treated samples were highly significant (P < 0.0001) in both variables (time and treatment, as well as the interaction between them) as measured by two-way ANOVA. Post-hoc analysis by the Bonferroni test revealed also significant differences between treatment and controls at times 9, 12 and 18 h (*P < 0.05; ***P < 0.001). (**G**) Immunoblots of aliquots removed at different times from cultures used in *panel* F before (−) and after treatment (+) with PK (3 μg/ml).

One of the hallmark properties of mammalian and yeasts prions is the formation of β-sheet rich amyloid-like structures by a seeding/nucleation mechanism^8^,^28^. Interestingly, the conversion of Mcc^a^ into Mcc^ia^ is also associated with the formation of amyloid-like structures, which bind to the amyloid-specific dyes: Thioflavin T (ThT) and Congo red (CR). Incubation of purified Mcc^a^ *in vitro* led to the formation of amyloid-like structures through a nucleation-dependent mechanism as illustrated by the ThT binding assay (Fig. 2A). The amyloid nature of the aggregates was further confirmed by a CR binding assay, in which CR bound to Mcc^ia^ displayed a characteristic increase in absorption and a spectral shift to reach a peak at ~540 nm compared to Mcc^a^ (Fig. 2B). Analysis by fourier-transformed infrared spectroscopy (FTIR) revealed a maximum absorption at ~1654 cm^−1^ for Mcc^a^, consistent with a predominance of α-helix/random coil structure. However, Mcc^ia^ exhibited the maximum absorption at ~1639 cm^−1^ indicating that β-sheets are the predominant structure (Fig. 2C)^29^. Further evidence for the structural differences between Mcc^a^ and Mcc^ia^ was the recognition with the conformational antibody A11. This antibody has been shown to specifically recognize β-sheet-rich oligomers produced during the process of amyloid formation of many different proteins^30^. Using a dot blot assay, we found that while Mcc^ia^ was readily recognized by A11, Mcc^a^ was not (Fig. 2D). Finally, while no detectable polymeric structures were seen for Mcc^a^ under electron microscopy, an aliquot of Mcc^ia^ was greatly enriched in unbranched amyloid-like fibrils of variable length (Fig. 2E).

To further investigate whether conversion of Mcc^a^ into Mcc^ia^ amyloid aggregates is the key event in changing the activity of the protein, we studied the effect of known and general inhibitors of amyloid formation on Mcc activity. For this purpose, we used curcumin and CR, which have been shown in several reports to prevent protein misfolding and aggregation into amyloid-like structures formed by diverse proteins^31^,^32^,^33^. Mcc producing bacteria were grown in the absence (control) or presence of either 25 μM curcumin or 50 μM CR. Aliquots of culture medium were subjected to Mcc activity assay and limited proteolysis. The addition of curcumin or CR significantly increased the time in which Mcc was active, suggesting an inhibitory effect of these molecules on the conversion of Mcc^a^ into Mcc^ia^ (Fig. 2F). This effect was not due to a change on bacterial growth, since under these conditions the rate of growth was the same in the presence or the absence of these molecules (data not shown). The influence of these molecules on the Mcc conformational change could be further monitored by studying the rate of proteolytic degradation of Mcc by PK. Both CR and curcumin completely inhibited the conversion of PK-sensitive Mcc^a^ into PK-resistant Mcc^ia^ until 12 h as compared to the control, where complete acquisition of PK-resistance was observed at that time point (Fig. 2G). Altogether, these results demonstrate that formation of Mcc^ia^ amyloid fibrils sequesters functional Mcc^a^ into the aggregates, leading to the loss of bacteriotoxin activity.

### The exogenous addition of culture medium containing Mcc^ia^ induces the prion-like conversion of Mcc^a^ into Mcc^ia^ *in vivo*

**Figure 3:**
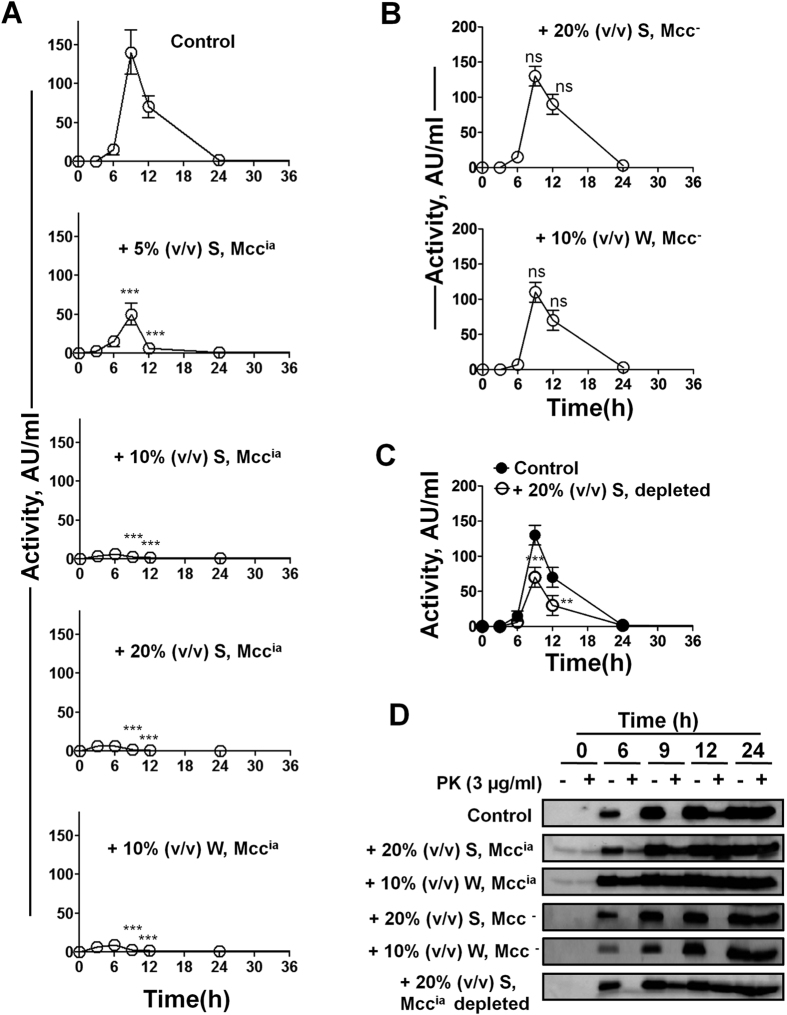
Exogenous addition of Mcc^ia^ induces the conversion of Mcc^a^ into Mcc^ia^ *in vivo*. (**A**) *E. coli* VCS257pJEM15 was grown in minimal medium at 37 °C until 36 h either in the absence (*control*) or in the presence of 5–20% (v/v) culture supernatant (S) or 10% (v/v) whole (W) culture containing Mcc^ia^. Aliquots were removed at indicated times and activity of Mcc was measured by CDM. The differences in Mcc activity between control and the various treatments were highly significant (P < 0.0001) in both variables (time and treatment, as well as the interaction between them) as measured by two-way ANOVA. Post-hoc analysis by the Bonferroni test revealed also significant differences between treatment and controls at times 9 and 12 h (***P < 0.001). (**B**) Mcc producing bacteria were grown in minimal medium in the presence of 20% (v/v) culture supernatant (S) and or 10% (v/v) whole (W) culture of *E. coli* VCS257 deprived of Mcc plasmid (Mcc^-^). Activity of Mcc was measured by critical dilution. In this case the differences between control and treatments were not significant as analyzed by two-way ANOVA. (**C**) Mcc producing bacteria were grown in minimal medium at 37 °C either in the absence (*control*) or in the presence of 20% (v/v) culture supernatant (S) after immunodepletion using an antibody specific for the sequence of Mcc. Aliquots were removed at indicated times and activity of Mcc was measured by CDM. In this experiment the treatment produced significant differences compared to the untreated control (used the same as panel A, top graph). Individual differences were analyzed by the Bonferroni post-test (*P < 0.05; **P < 0.01; ***P < 0.001). In panels A, B and C, activity was measured in duplicate samples and *error bars* indicate S.D. **D**: Immunoblots of aliquots removed from culture used in *panels* A, B and C before (−) after treatment (+) with PK (3 μg/ml) at indicated time points.

The hallmark feature of prions is that the pathological, infectious form (PrP^Sc^) interacts with endogenous PrP^C^ and catalyzes its conformational change to produce more PrP^Sc^. To test whether Mcc^ia^ is capable of catalyzing the Mcc^a^ to Mcc^ia^ conversion, Mcc producing bacteria were grown in minimal media at 37 °C in the absence (control) or in the presence of 10% (v/v) whole culture or 5–20% (v/v) culture supernatant, both taken from the stationary phase of the culture when Mcc^ia^ is naturally produced (see “Materials and Methods”). Small aliquots were periodically removed and subjected to the Mcc activity assay by CDM and proteolysis by PK (3 μg/ml). As expected, the aliquots from Mcc producing bacteria grown in the absence of Mcc^ia^ showed maximum activity at 9 h that started to decline at 12 h, and was undetectable by 24 h (Fig. 3A). The loss of activity correlated with the appearance of PK-resistant Mcc at 12 h, whereas Mcc was completely sensitive to PK until 9 h (Fig. 3D). The addition of 5 to 20% (v/v) culture supernatant containing Mcc^ia^ significantly diminished the amount of active Mcc present in the bacteria culture in a dose-dependent manner, reaching a complete effect when 10% of the material was added (Fig. 3A). Consistently, the loss of activity was paralleled with the production of PK-resistant Mcc (Fig. 3D). To exclude the possibility that the loss of Mcc activity after addition of bacterial culture medium was due to another factor present in the medium rather than the prion-like activity of Mcc^ia^, we conducted a control experiment in which culture medium from the stationary phase of bacteria devoid of Mcc plasmid was exogenously added. The addition of either whole culture [10% (v/v)] or culture supernatant [20% (v/v)] into Mcc producing bacteria had no significant effect on Mcc activity (Fig. 3B) or in the appearance of PK-resistant Mcc (Fig. 3D), as compared to the control. To further show that Mcc^ia^ promotes the conversion of Mcc^a^ into Mcc^ia^, we depleted Mcc^ia^ from the medium by using a Mcc-specific antibody. Unfortunately, immunodepletion only partially removed Mcc^ia^ from the samples, as determined by immunoblotting (Supplementary Figure 1). Nevertheless, the addition of culture supernatant containing Mcc^ia^ [20% (v/v)] after immunodepletion partially prevented the inactivation of the bacteriotoxin (9 h, P < 0.001; 12 h, P < 0.01; Fig. 3C), which was also paralleled by the partial reduction of acquisition of PK resistance (Fig. 3D, bottom panel). These results suggest that Mcc^ia^ released by bacteria at the stationary phase of the culture is able to catalyze the conversion of Mcc^a^ into Mcc^ia^, leading to the permanent loss of bacteriotoxin activity by changing the phenotype of the bacteria.

The results shown above indicate that the conversion of Mcc^a^ into Mcc^ia^ is dependent on the presence and concentration of Mcc^ia^ seeds. Next, we sought to investigate the optimum time for the addition of exogenous Mcc^ia^. For this purpose, we added 20% (v/v) culture supernatant containing Mcc^ia^ at different time points (0, 3, and 6 h) during the growth phase of Mcc producing bacteria (Supplementary Figure 2A). The addition of Mcc^ia^ until 3 h of the growth phase, completely abolished Mcc activity, however, when added at 6 h it was only partially effective in catalyzing Mcc^a^ into Mcc^ia^ conversion (P < 0.001; Supplementary Figure 2B).

Another important feature of prions is that once PrP^Sc^ is formed, it can self-perpetuate indefinitely by serial passages in infected animals. Thus, we wanted to explore whether this also happens with Mcc^ia^ after its first cycle of conversion. For this purpose, Mcc producing bacteria were grown until 48 h (stationary phase; P0). After 48 h, 10% (v/v) of whole culture was added into the fresh culture medium of Mcc producing bacteria and grown for a subsequent 48 h (P1). This cycle was repeated up to 3 passages (Supplementary Figure 3A). Aliquots from all passages were removed, and Mcc activity was analyzed as described before. In P0, where no Mcc^ia^ was added, Mcc activity showed a typical trend; increase and then decrease in activity at the stationary phase. However, in all passages P1 to P3, Mcc activity was undetectable at any time point (Supplementary Figure 3B), suggesting that after the first conversion of Mcc^a^ into Mcc^ia^, Mcc^ia^ propagates its conformation by catalyzing Mcc^a^ conversion (Supplementary Figure 3B). This experiment shows that the presence of small amounts of Mcc^ia^ effectively and permanently changes the phenotype of the bacteria culture from able to unable to induce the death of competing bacteria.

### The exogenous addition of *in vitro*-generated, purified Mcc^ia^ induces the prion-like conversion of Mcc^a^ into Mcc^ia^ *in vivo*

**Figure 4:**
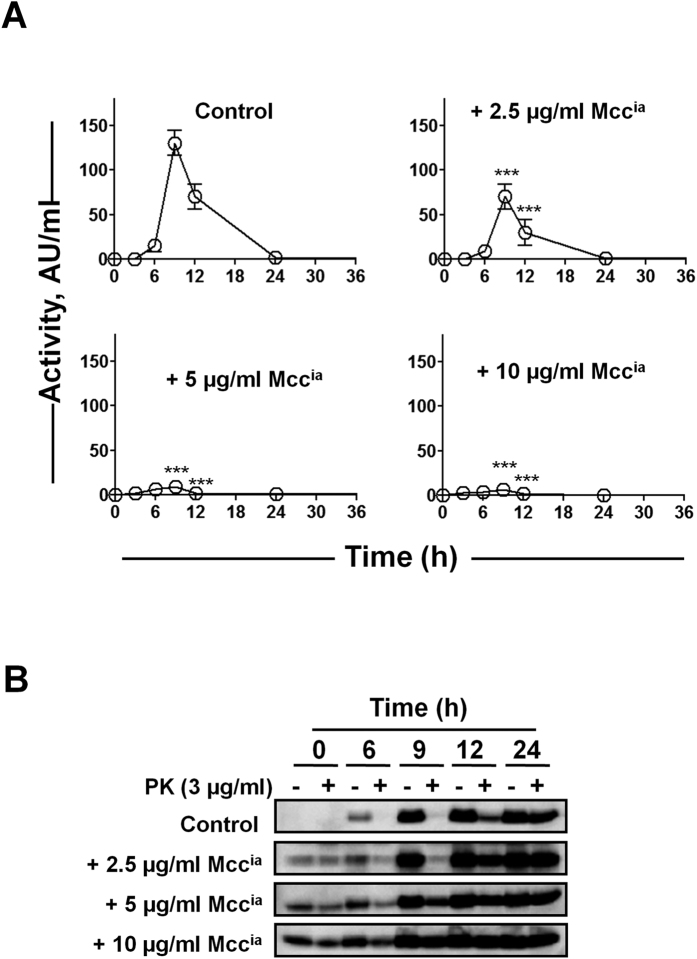
Purified *in vitro*-generated Mcc^ia^ induces the conversion of Mcc^a^ into Mcc^ia^ *in vivo*. (**A**) *E. coli* VCS257pJEM15 was grown in minimal medium at 37 °C either in the absence (*control*) or in the presence of purified Mcc^ia^ (2.5, 5 and 10 μg/ml, final concentration; see “Materials and Methods”). Aliquots were removed at indicated time points and Mcc activity was measured by CDM. Activity was measured in duplicate samples and *error bars* indicate S.D. The differences in Mcc activity between control and treated samples were highly significant (P < 0.0001) in both variables (time and treatment, as well as the interaction between them) as measured by two-way ANOVA. Post-hoc analysis by the Bonferroni test revealed also significant differences between treatment and controls at times 9 and 12 h (***P < 0.001). (**B**) Immunoblots of aliquots removed from culture used in *panel A* before (−) and after treatment ( + ) with PK (3 μg/ml) at indicated time points.

To provide evidence that conversion of Mcc^a^ into Mcc^ia^ *in vivo* is a protein-only phenomenon, similar to what has been shown for mammalian and yeast prions, we studied whether *in vitro* produced Mcc^ia^ aggregates can catalyze Mcc conversion in the bacterial culture. For this purpose, highly purified Mcc^a^ (400 μg/ml) was allowed to aggregate into Mcc^ia^ *in vitro* in minimal medium at 37 °C for 48 h with vigorous shaking. After 48 h, the amyloid formation was confirmed by a ThT binding assay (data not shown), and the *in vitro* generated Mcc^ia^ was used to induce conversion of Mcc^a^ into Mcc^ia^ *in vivo*. Mcc producing bacteria were grown in the absence (control) or presence of various concentrations of Mcc^ia^ (2.5–10 μg/ml) for 48 h at 37 °C. Aliquots were removed at different time points and subjected to Mcc activity assay and proteolysis by PK. The addition of Mcc^ia^ significantly diminished the activity of Mcc produced by bacteria in a dose-dependent manner (P < 0.001; Fig. 4A). No detectable bacteriotoxin activity was observed in the presence of 5 or 10 μg/ml of Mcc^ia^, whereas a lower concentration of Mcc^ia^ (2.5 μg/ml) was only partially effective (Fig. 4A). Consistently, the loss of activity of Mcc correlated with the emergence of PK-resistant Mcc as shown by immunoblot analysis (Fig. 4B). This result clearly indicates that Mcc^ia^ prepared *in vitro* from highly purified Mcc^a^ in the absence of any other factors is capable of self-propagating in culture by catalyzing the conversion of endogenous Mcc^a^ into Mcc^ia^.

### Prion-like activity of Mcc^ia^ is conformation dependent

**Figure 5:**
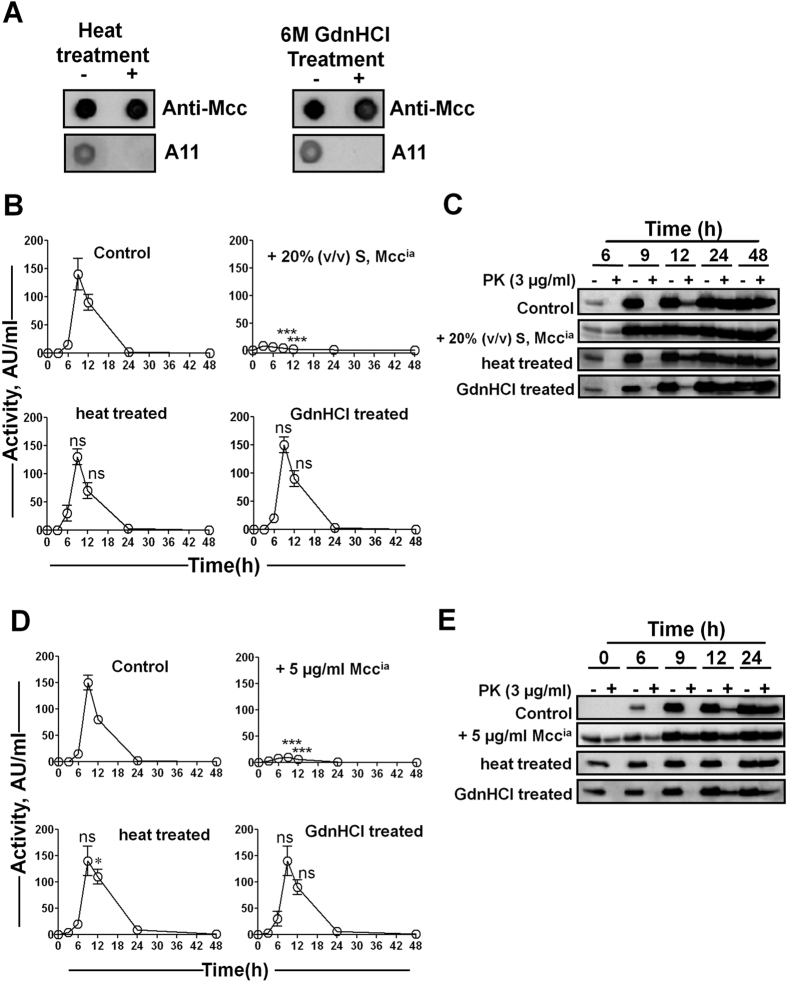
Mcc^ia^ prion-like activity is conformation dependent. (**A**) To analyze whether heat and chemical denaturation altered Mcc^ia^ conformation, purified Mcc^ia^ was subjected to heat or GdnHCl treatment as indicated in “Materials and Methods” and reactivity with the A11 conformational antibody was studied. Samples were spotted onto nitrocellulose membrane and probed with the A11 oligomer specific antibody and anti-Mcc antibody. (**B**) *E. coli* VCS257pJEM15 was grown in minimal medium at 37 °C either in the absence (*control*) or in the presence of 20% (v/v) culture supernatant (S) that was untreated, treated with heat, or treated with GdnHCl. Aliquots were removed at indicated times and Mcc activity was measured by CDM. **C**: Immunoblots of aliquots removed from culture used in *panel B* before (−) and after treatment (+) with PK (3 μg/ml) at indicated time points. (**D**) Similarly, Mcc producing bacteria were grown in minimal medium either in the absence (*control*) or in the presence of purified Mcc^ia^ (5 μg/ml) that was untreated, treated with heat, or treated with GdnHCl. Aliquots were removed at indicated times and activity of Mcc was measured by CDM. In experiments shown in *panels B and D* activity was measured in duplicate samples and *error bars* indicate S.D. **E**: Immunoblots of aliquots removed from culture used in *panel D* before (−) and after treatment (+) with PK (3 μg/ml) at indicated time points. The differences in Mcc activity between control and treated samples at 9 and 12 h in *panels B* and D were analyzed by two-way ANOVA with Bonferroni post-test (ns, P > 0.05; *P < 0.05; ^#^P < 0.001).

Although the prion activity of mammalian and yeast prions is resistant to harsh procedures that destroy nucleic acids, it is sensitive to agents that alter protein conformation^34^. To gain insight into whether the conversion of Mcc^a^ into Mcc^ia^ is conformation dependent, Mcc producing bacteria were exposed to Mcc^ia^ untreated or subjected to heat or chemical denaturation. For this purpose, Mcc^ia^ seeds either produced *in vivo* or generated *in vitro* were denatured by boiling for 15 min, or by incubating with 6 M guanidine hydrochloride (GdnHCl) for 2 h at room temperature (see “Materials and Methods”). Both procedures disrupted the conformation of Mcc^ia^, as evaluated by dot blot using the A11 conformational antibody (Fig. 5A). However, Mcc could be easily detected by an antibody specific for the C-terminal sequence of Mcc.

Mcc producing bacteria were grown in the absence (control) or presence of culture supernatant containing Mcc^ia^ [20% (v/v)] or *in vitro*-generated, purified Mcc^ia^ (5 μg/ml) as such or after denaturation either by boiling or by treatment with 6 M GdnHCl. Aliquots from the culture were periodically removed, and Mcc activity and its resistance to PK were tested. The addition of untreated purified, *in vitro*-generated Mcc^ia^ or culture containing Mcc^ia^ consistently resulted in loss of Mcc activity and early formation of PK resistant Mcc (Fig. 5B–E); however, the addition of purified Mcc^ia^ or culture containing Mcc^ia^ after exposure to boiling and to GdnHCl had no effect either on Mcc activity or on the formation of the PK resistant Mcc as compared to the control (Fig. 5B–E). These results suggest that destruction of Mcc^ia^ conformation abolishes its ability to self-propagate and induce the phenotypic changes to the bacterial culture.

### Mcc behaves as a prion in a yeast prion reporter assay

**Figure 6:**
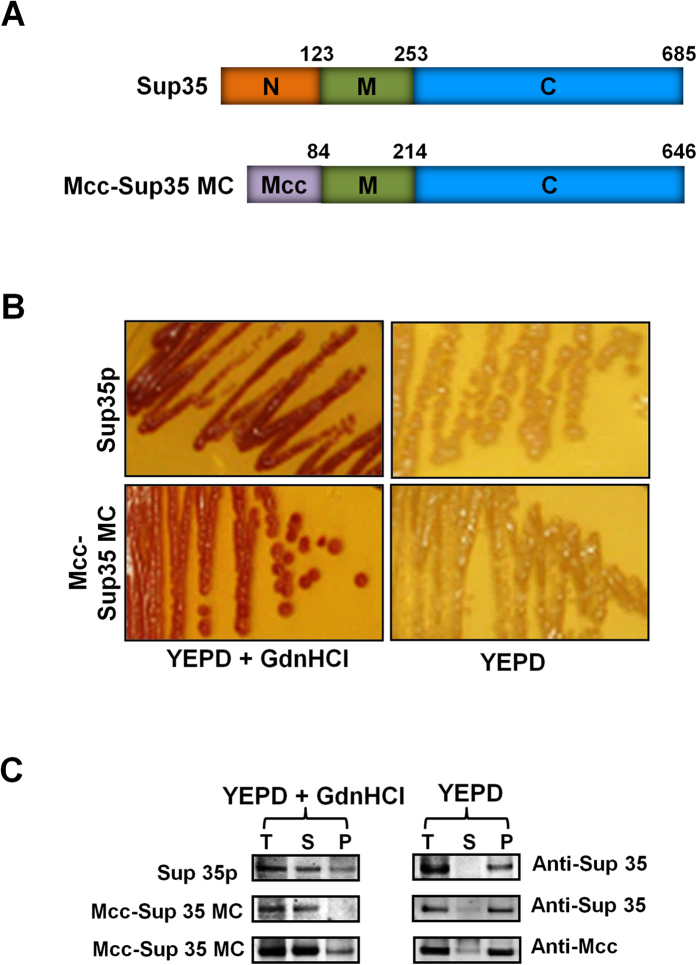
Mcc confers prion behaviors to Sup35 in a prion reporter assay in yeast. (**A**) Schematic representation of full length Sup35p containing the N, M and C domains and Mcc-Sup35MC fusion protein containing the full-length mature Mcc sequence (1–84) instead of its endogenous N region prion-forming domain (1–123 amino acids). (**B**) The color based assay that measures distinct heritable phenotypes of Sup35p and Mcc-Sup35MC fusion protein are shown after cells expressing Sup35p (*as a positive control*) and Mcc-Sup35MC fusion protein were plated onto YEPD and YEPD plates supplemented with 3 mM GdnHCl. (**C**) The sedimentation analysis of total cell extracts of cells expressing Sup35p and Mcc-Sup35MC grown on either YEPD alone or on YEPD supplemented with 3 mM GdnHCl. Total cell extracts (T) were centrifuged at 40,000 rpm for 30 min, resulting in supernatant (S) and pellet (P) that were separated on SDS-PAGE, immunoblotted, and probed with anti-Mcc and anti-Sup35p antibodies.

To further explore the prion-like behavior of Mcc, we used a well-established prion reporter assay in yeast, based on [*psi*^-^] and [*PSI*^+^] states of the translation termination factor Sup35p^35^. The prion domains in yeast prions including Sup35p are modular and can transfer the prion behaviors to heterologous proteins^36^. We used this feature of yeast prions to generate Mcc-Sup35MC fusion protein, by replacing the N-terminal prion domain of Sup35 with full-length mature Mcc which was fused to the functional MC domain of Sup35p (see “Materials and Methods” for details) (Fig. 6A). The fusion protein was tested for heritable [*PSI*^+^] state using the *ADE1* gene containing a premature stop codon. In [*PSI*^+^] cells, Sup35p is assembled into an inactive self-perpetuating prion form which does not take part in premature translation termination and thus cells are able to grow on adenine-deficient medium and produce white colonies (Fig. 6B). Whereas in the case of [*psi*^−^] state, cells do not make functional Ade1, and red colonies appear due to the accumulation of red by-product. Surprisingly, like other yeast prions, Mcc conferred prion behavior to Sup35MC. Indeed, cells expressing Mcc-Sup35MC fusion protein gave rise to white colonies on YEPD plates, which were maintained after repeated streaking (Fig. 6B) in a similar manner as Sup35p in the [*PSI*^+^] state (positive control). The propagation of a prion phenotype for all known yeast prions is dependent on the activity of the heat shock protein 104 (Hsp104)^37^; therefore, yeast prions can be cured by inhibiting Hsp104 either by chemical inhibition or by genetic manipulation^38^,^39^. Similar to Sup35p (used as a positive control), the prion form of Mcc-Sup35MC fusion protein could also be cured by transferring a white colony to YEPD plates containing 3 mM of GdnHCl, a known inhibitor of Hsp104 (Fig. 6B). These results clearly suggest that Mcc confers to Sup35MC the ability to exist in two distinct physical and functional states, which are interconvertible and heritable by the prion mechanism.

To further confirm the prion behavior of Mcc-Sup35MC in yeasts, we studied the formation of aggregates by a sedimentation assay. The prion form exists in high assemblies and can be pelleted by high-speed centrifugation, whereas non-prion forms mostly exist in a soluble state^40^. The white colonies expressing Mcc-Sup35MC or Sup35p (positive control) were grown in YEPD medium, whereas red colonies from Mcc-Sup35MC or Sup35p were grown in YEPD medium supplemented with 3 mM GdnHCl. The total cell extracts (T) were fractionated by high-speed centrifugation, and supernatant (S) and pellet (P) fractions were probed with anti-Mcc as well as with anti-Sup35p. Most of the Mcc-Sup35MC fusion protein was present in an insoluble form when cells were grown in YEPD, which indicates formation of large assemblies similar to Sup35p in [*PSI*^+^] state. However, when cells were grown on YEPD plates containing 3 mM GdnHCl, most of the Mcc-Sup35MC fusion protein was present in the soluble fraction, as the case for Sup35p in the [*psi*^−^] state (Fig. 6C). These results clearly suggest that change in heritable phenotype is, indeed, due to the distinct physical states of Mcc-Sup35MC fusion protein in yeast.

### Identification of the Mcc prion domain (PrD)

**Figure 7:**
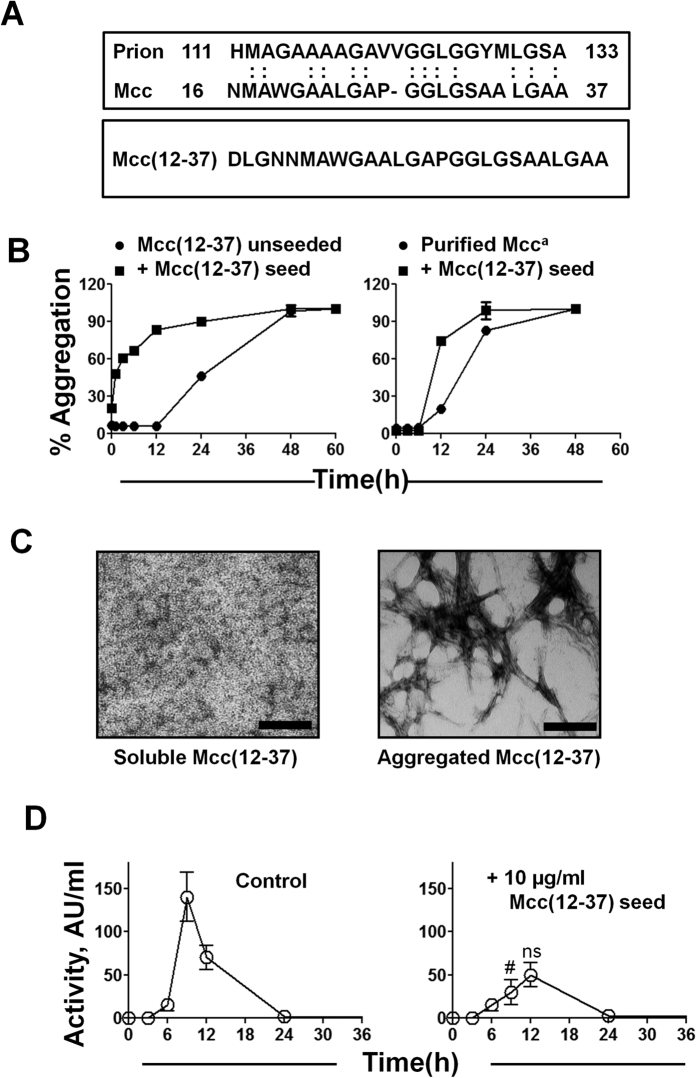
Identification of a prion domain in Mcc with sequence similarity to human PrP. (**A**) Sequence similarity between mature Mcc (residues 16–37) and human PrP (amino acids 111–133) (*upper panel*). Amino acid sequence of a synthetic peptide spanning residues 12–37 of mature Mcc used to model the PrD. (**B**) Synthetic Mcc(12-37) (228 μg/ml) and purified soluble Mcc^a^ (400 μg/ml) were incubated in the absence (⦁) or in the presence (▪) of preformed aggregates (seeds) of Mcc(12-37) (11 μg/ml) in aggregation buffer until 60 h at 37 °C. (**C**) The morphological analysis by TEM of soluble Mcc(12-37) (*left panel*) and aggregated Mcc(12-37) (*right panel*). The bars represent 100 nm. (**D**) Mcc producing bacteria were grown in minimal medium at 37 °C either in the absence (*control*) or in the presence of preformed Mcc(12-37) aggregates. Aliquots were removed at indicated times and Mcc activity was measured by CDM. Activity was measured in duplicate samples and *error bars* indicate S.D. The differences in Mcc activity between control and treated samples at 9 and 12 h were analyzed by two-way ANOVA with Bonferroni post-test (ns, P > 0.05; ^#^P < 0.001).

To study the region of Mcc responsible for its prion-like activity, we compared the sequence of Mcc with that of human PrP. As shown in Fig. 7A, a remarkable sequence similarity was found between the central region of mature Mcc (amino acids 16–37) with the internal hydrophobic core region of PrP (amino acids 111–133). Indeed, a 57% sequence identity and a 74% similarity was observed in these regions between these proteins from two greatly distant organisms. Importantly, various pieces of evidence suggest that this region of PrP plays an important role in the formation of PrP^Sc^ (refs 41 and 42). To analyze whether this region of Mcc might be the domain responsible for prion activity, we investigated if this sequence can form self-replicating amyloid aggregates that can induce the conversion of full-length Mcc^a^ into Mcc^ia^ *in vitro* and *in vivo*. For this purpose, we used a synthetic peptide comprising the residues 12–37 of mature Mcc. The reason for using this sequence instead of Mcc (16–37) was to increase the peptide solubility and easiness to handling. To examine whether Mcc(12-37) forms amyloid aggregates *in vitro,* we performed a ThT binding assay and analyzed the aggregates’ morphology by electron microscopy. Purified synthetic Mcc(12-37) at a concentration of 228 μg/ml was allowed to aggregate and aliquots were tested for ThT binding. As shown in Fig. 7B, Mcc(12-37) formed amyloid following a nucleation-dependent mechanism, characterized by a 12 h lag phase. Interestingly, preformed aggregates of Mcc(12-37) were capable of seeding the aggregation of both soluble Mcc(12-37) and full-length purified Mcc^a^, reducing their lag phase (Fig. 7B). The amyloid nature of Mcc(12-37) aggregates was confirmed by transmission electron microscopy (Fig. 7C).

We next examined whether Mcc(12-37) aggregates are able to induce the conversion of Mcc^a^ into Mcc^ia^ *in vivo*. Preformed Mcc(12-37) aggregates (10 μg/ml) were exogenously added to a fresh culture of Mcc producing bacteria, and bacteriotoxin activity was determined by CDM. The addition of exogenous Mcc(12-37) seeds *in vivo* partially inhibited the activity of Mcc which is indicative of induced conversion of Mcc^a^ into Mcc^ia^ (Fig. 7D). These results clearly suggest that the region spanning residues 12-37 of Mcc, sharing similarity with the central region of PrP, might be the prion-forming domain of Mcc.

## Discussion

The capability of proteins to self-propagate and transmit biological information in a similar manner as genetic material is a recently recognized concept. Prions were first identified as proteinaceous infectious agents responsible for various catastrophic neurodegenerative disorders known as prion diseases or TSEs^4^. In TSEs, a naturally occurring protein undergoes conformational changes leading to the formation of a misfolded and aggregated form, termed PrP^Sc^. Like a typical infectious micro-organism, PrP^Sc^ can be transferred from individual-to-individual by various routes of administration and replicate in the new host forming more PrP^Sc^. PrP^Sc^ self-replication involves the catalytic conversion of the normal isoform of the prion protein (PrP^C^) through a seeding/nucleation mechanism, in which the PrP^Sc^ aggregate binds PrP^C^ and promotes its misfolding by incorporation into the growing polymer^8^. Initially, the prion phenomenon was thought to be exclusive for TSEs, but in recent years several other neurological and systemic disorders have been proposed to spread by the prion principle^8^. More importantly, the prion mechanism of transmission of biological information by propagation of protein conformational changes has been shown not to be restricted to diseases, but to operate in diverse organisms to modulate the biological function of certain proteins^8^,^17^. The seminal discovery of self-perpetuating prion proteins in yeast and other fungus has proven that prion-like conformational changes can also have a beneficial outcome^16^. Unlike prion protein, yeast prions are generally nonpathogenic and produce distinct phenotypes with different physiological functions, providing a competitive advantage in adverse conditions^11^. Later, several other beneficial prions have been discovered that use prion-like conformational changes to propagate biological signals. For example, the self-perpetuating fiber-like polymers of MAVS regulate mammalian antiviral immune defense^19^ and a translation regulator of the invertebrate *Aplysia* forms self-perpetuating polymers that help to maintain long-term potentiation in sensory neurons^18^. These findings clearly suggest that diverse organisms harness prion-like conformational changes in several unrelated proteins to regulate biological function. However, it is currently unclear how frequent and universal the use of the prion principle is for modulating the biological activity of proteins.

Here, we report a bacterial protein that exhibits prion-like characteristics and could be considered as the first example of a prokaryotic prion. Mcc possesses several hallmark features of authentic prions, namely: (1) The protein exists in two structurally and functionally different forms, Mcc^a^ and Mcc^ia^; (2) Mcc^ia^ is the prion form that adopts a β-sheet-rich amyloid-like conformation that is formed by a seeding/nucleation mechanism; (3) Like PrP^Sc^, Mcc^ia^ is highly resistance to digestion by large concentrations of proteases, whereas Mcc^a^ (like PrP^C^) is readily digested; (4) Exogenous administration of Mcc^ia^ to the culture of Mcc-producing bacteria induces the rapid conversion of Mcc^a^ into Mcc^ia^, leading to the loss of bacteriotoxin activity and the change of the phenotype of the bacteria; (5) Like PrP^Sc^, Mcc^ia^ can be maintained self-propagating *in vivo* by multiple passages of experimental infection; (6) The prion-like activity of Mcc^ia^ can be abolished by treatments that destroy protein conformation and by inhibitors of amyloid formation; (7) The conversion of Mcc^a^ to Mcc^ia^ can be reproduced *in vitro* using purified protein, and the *in vitro*-generated Mcc^ia^ is able to self-replicate *in vivo* and change the bacterial phenotype; (8) Mcc can replace the PrD of Sup35p, and induce [PSI^+^] phenotype in yeasts when fused to the functional domain of Sup35p. However, in contrast with mammalian prions, the prion form of Mcc is not toxic and the non-prion form is the toxic one. This apparent discrepancy may not be so, because evidence accumulated in the past several years have shown that the formation of large amyloid aggregates in TSEs as well as other amyloid-related disorders (e.g. Alzheimer’s or Parkinson’s diseases) may actually be a protective mechanism to decrease the amount of small oligomers which are the real toxic species^43^. Another contrasting feature that differentiates Mcc from other prions is that Mcc^ia^ is located in the extracellular space and thus the conformational information inherent to Mcc is dissociated from the organism and not passed on to neighboring or daughter cells. Because of this, the biological changes are not transmitted to another organism, but to the environment in which bacteria live. Currently, the definition of prions is changing in the field thanks to many reports showing prion-like properties of various other proteins. Thus, at this time is yet unclear whether prions need to be associated to cells or changing the biological properties of cells upon transmission. For example, many reports have provided evidences for the Alzheimer’s amyloid-beta protein behaving like a prion^44^,^45^,^46^, and this is also an extracellular protein which arguably changes the environment in the brain (not necessarily the cells). Bacteria living in the gut and releasing Mcc into the medium may produce a very similar result as amyloid-beta in the brain of Alzheimer’s patients.

One of the surprising features of the Mcc prion behavior is its sequence similarity with the central region of PrP. The sequence 16–37 of Mcc shows 57% identity and 74% similarity with the PrP sequence spanning residues 111–133 (Fig. 7A). Various pieces of evidence have shown that this region of PrP plays an essential role in PrP conversion and its pathological properties, including: (a) The synthetic peptide comprising the sequence 106–126 of PrP has been widely used to model prion neurotoxicity^47^,^48^,^49^; (b) Deletion of the fragment 114–121 results in a protein that cannot be converted into PrP^Sc^ (ref. 42); (c) Synthetic peptides spanning the sequence 109–141 of PrP undergo conformational changes that mimic the conversion of PrP^C^ into PrP^Sc^ (ref. 50); (d) Synthetic peptides harboring the sequence 109–141 or 106–128 of PrP can effectively inhibit the interaction of PrP^C^ and PrP^Sc^ (ref. 50); (e) The region 115–130 has been used to produce β-sheet breaker peptides able to reverse the PrP^Sc^ pathological conformation and reduce infectivity *in vivo*^51^; (f) The central region of PrP is highly conserved among mammals and many of the pathological mutations linked with inherited TSEs are located in this region^3^. The similarity of Mcc with this important region of PrP suggests that this sequence could be key to the prion properties of both PrP and Mcc. Indeed, synthetic Mcc(12-37) forms amyloid fibrils through a nucleation-dependent mechanism and is able to seed itself and full-length Mcc *in vitro*. Moreover, aggregates of Mcc(12-37) can induce the conversion of Mcc^a^ into Mcc^ia^ *in vivo*. More experiments are needed to determine the minimum sequence of the Mcc prion domain.

Our findings indicate that Mcc exhibits several characteristics of prions; however, the functional relevance of the Mcc prion mechanism is unclear. Mcc producing bacteria secrete Mcc^a^ that kills competing bacteria, helping them to occupy a spatial niche in a given ecosystem. However, when Mcc producing bacteria have prevailed, Mcc^a^ is no longer needed. We can speculate that under these conditions, the prion-like mechanism may operate to encapsulate Mcc^a^ into Mcc^ia^, protease resistant aggregates that may serve as a reservoir for Mcc^a^. Later if the conditions of the ecosystem change and competition arise, Mcc^ia^ aggregates may undergo conformational changes that release Mcc^a^ thus providing a competitive advantage. We have previously shown that Mcc^ia^ amyloid aggregates can indeed release Mcc^a^ upon changing environmental conditions^52^. In summary, our findings indicate that the prion mechanism is not restricted to eukaryotic organisms, but is likely an ancient process of regulation of protein function by rapid self-propagation of changes in protein conformation. Thus, the prion principle is a conserved process occurring all the way from bacteria to humans.

## Materials and Methods

Chemicals were obtained from Sigma (St. Louis, Mo), unless otherwise stated.

### Purification of Microcin E492 (Mcc)

Mcc was purified from the culture supernatants as described earlier^27^. In brief, *E. coli* VCS257 cells harboring pJEM15 plasmid were grown in M9 minimal medium containing 0.2% glucose, 0.2% sodium citrate, 1 g/liter casamino acid, 1 mg/liter thiamine and 100 mg/liter ampicillin to an optical density of 1.2 at 600 nm at 37 °C with shaking. Bacterial cells and debris were removed by centrifugation at 4000 rpm for 10 min. The resultant supernatant was passed through a Sep-Pak C18 cartridge (Waters). The cartridge was sequentially washed with 65% methanol, and 25% acetonitrile. Finally, the bound Mcc was eluted with 50% acetonitrile, and lyophilized. Lyophilized powder of Mcc (purified active Mcc) was stored at −20 °C until used. Under these conditions, the preparation contains highly purified Mcc ( > 90%) as evaluated by silver stained gels^53^.

### Aggregation of purified Mcc^a^ and synthetic Mcc(12-37), and seeding *in vitro*

To start Mcc aggregation, purified Mcc^a^ powder was dissolved in sterile 10 mM NaOH solution, and filtered through a 30 kDa cutoff filter to remove aggregates. The fragment 12-37 of Mcc was synthesized in solid phase and purified by reverse phase chromatography. Lyophilized powder of synthetic Mcc(12-37) was dissolved in 0.1% NH_4_OH at a concentration of 2 mg/ml, centrifuged at 16,500 × g for 10 min to remove any aggregates. Purified Mcc^a^ (400 μg/ml) and soluble Mcc(12-37) (228 μg/ml) were incubated in aggregation buffer (50 mM PIPES-NaOH, pH 6.5, 0.5 M NaCl) for 48 h at 37 °C with vigorous shaking. For seeding of Mcc(12-37) and full length Mcc^a^ *in vitro*, 48 h aggregated Mcc(12-37) was used as a seed. Mcc^a^ (400 μg/ml) and Mcc(12-37) (228 μg/ml) were incubated in aggregation buffer in the absence or in the presence of pre-aggregated Mcc(12-37) seed (11 μg/ml) until 60 h at 37 °C with vigorous shaking.

### Preparation of purified Mcc^ia^ and culture containing Mcc^ia^ for seeding *in vivo*

To isolate Mcc^ia^ from the culture of Mcc producing bacteria, a 1:1000 dilution of overnight culture of Mcc producing bacteria was allowed to grow for 48 h, until reaching the stationary phase, at 37 °C with shaking. After 48 h, either whole bacterial culture (W) or culture supernatant (S) after removing bacterial cells by centrifuging at 4000 rpm for 10 min was used for seeding *in vivo* (Supplementary Figure 4). Mcc^ia^ can also be produced *in vitro* by inducing the aggregation of Mcc^a^, as described before.

### Denaturation of Mcc^ia^

To denature Mcc^ia^, purified Mcc^ia^ prepared *in vitro* and culture supernatant containing Mcc^ia^ were either boiled for 15 min or incubated with 6 M GdnHCl for 2 h at room temperature followed by dialysis to remove GdnHCl, and used for *in vivo* seeding.

### Thioflavin T (ThT) binding assay

The kinetics of Mcc aggregation was monitored by ThT binding assay, as described^54^. Mcc (400 μg/ml) and Mcc(12-37) (228 μg/ml) were allowed to aggregate in 50 mM PIPES (pH 6.5) containing 500 mM NaCl at 37 °C with vigorous shaking for different times. At the end of the reaction, 20 μl of the mixture was mixed with 80 μl of 6 μM ThT in 1X PBS (Phosphate Buffer Saline), and fluorescence was measured immediately at excitation of 435 nm and emission of 485 nm, respectively, using a microplate spectrofluorometer Gemini-EM (Molecular Devices, Sunnyvale, CA).

### Transmission electron microscopy (TEM)

An aliquot of 10 μl of reaction sample was placed onto Formvar-coated 200-mesh copper grids for 5 min, washed at least three times with distilled water, and then negatively stained with 2% uranyl acetate for 1 min. Grids were examined by electron microscopy (H-7600, Hitachi, Japan) operated at an accelerating voltage of 80 kV.

### Congo red (CR) binding

Amyloid-like nature of Mcc^ia^ was examined by binding of the amyloid specific dye Congo red by the spectroscopic band shift assay^27^. Purified Mcc^a^ was allowed to aggregate in 50 mM PIPES (pH 6.5) containing 500 mM NaCl at 37 °C with vigorous shaking for 24 h. An aliquot was incubated with 12.5 μM CR for 30 min at 37 °C. The absorbance spectrum was recorded from 400 nm to 700 nm.

### Proteinase K (PK) digestion and Immunoblotting

Samples were incubated with the indicated concentrations of PK at 37 °C for 30 min. The reaction was stopped by boiling of the sample in NuPAGE LDS buffer at 100 °C for 10 min. Then, samples were resolved by NuPAGE 4–12% Bis-Tris gels (Invitrogen). Proteins were electrophoretically transferred to nitrocellulose membranes (Amersham Biosciences, Germany). Membranes were blocked with 5% w/v nonfat dry milk in Tris-buffered saline-Tween 20 (TBS-T, 20 mM Tris, pH 7.2, 150 mM NaCl and 0.05% (v/v) Tween 20) at room temperature for 2 h. After blocking, the membranes were probed with anti-Mcc antibody (1:3000) and the anti-rabbit horseradish peroxidase-conjugated secondary antibodies (1:5000). The blots were visualized using enhanced chemiluminescence plus western blotting detection kit (Amersham Biosciences, Piscataway, NJ).

### Dot blot analysis

Five μl of each reaction was spotted onto nitrocellulose membranes (Amersham Biosciences, Germany), and air dried for 30 min at room temperature. Finally, blots were blocked and visualized as described above (see PK digestion and Immunoblotting).

### Mcc activity assay

Activity of Mcc in each sample was measured by critical dilution method (CDM) as described previously^55^. In brief, samples were centrifuged at 16,500 × g for 10 min at room temperature. The resultant supernatant was serially diluted in minimal medium, and 5 μl aliquots were laid onto Luria Broth (LB) agar plates overlaid with Mcc-sensitive *E.coli* BL21 (DE3) p11α2 cells. After 16 h of incubation at 37 °C, the activity of Mcc in the last dilution that gave detectable inhibition (approximately 2-times above the background levels) was determined and expressed in arbitrary units/ml^55^.

### Immunodepletion of Mcc^ia^

Dynabeads sheep anti-rabbit antibody (Life Technologies, Oslo, Norway) were conjugated with anti-Mcc antibody overnight at 4 °C as per manufacturer’s instructions. Following conjugation, beads were washed and incubated with culture supernatant containing Mcc^ia^ overnight at 4 °C, beads were removed with a magnet and the resulting supernatants were used for seeding.

### Fourier-Transform Infrared spectroscopy (FTIR)

FTIR experiments were conducted in an FT/IR-4100 spectrometer from JASCO. Purified Mcc^a^ (400 μg/ml) was used as such or aggregated for 24 h, as described previously. Protein slurry was then placed on the top of a diamond PRO450-S Attenuated Total Reflectance unit from JASCO adapted to the FT/IR-4100 system. System parameters included 4.0 cm^−1^ resolution and an accumulation of 80 scans per sample. The data was processed using Cosine anodization and Mertz phase correction. The data was also corrected for ATR and carbon dioxide vapor absorption. Data fitting and secondary structure calculations of samples were analyzed, after buffer subtraction, by the Secondary Structure Estimation 4000 software from JASCO. Each sample was measured in triplicates to confirm the secondary structure.

### Yeast strain and Plasmid construction

To construct pJ528-MCC (*Leu*), the Microcin open rading frame was obtained by a polymerase chain reaction (PCR) using pJEM15 plasmid harboring Mcc gene, as the template, 5′ GCGCGGACGTCATGGGAGAGACC GATCCAAATACT 3′ as the forward primer, and 5′ GCGCGggatccCTACCACTACC GGAACTGG 3′ as the reverse primer. After digestion with AatII and BamHI, the PCR fragment was ligated to pJ528 (*Leu*) that was predigested with AatII and BamHI. Yeast strain 780-1D (*MATα kar1-1 SUQ5 ade2-1 his3 leu2 trp1 ura3 sup35*) with the *SUP35* maintainer plasmid pJ533 (*URA3*) was transformed with pJ528-MCC. The resulting transformants were grown on media containing 5-fluoroorotic acid to eliminate pJ533 (URA3). pJ528 (Leu), pJ533, and 780-1D were kindly provided by Dr. Dan Masison.

### Sedimentation assay

A single colony of each strain expressing Sup35p or Mcc-Sup35MC was inoculated in 3 ml liquid YEPD alone or supplemented with 3 mM GdnHCl, and grown overnight at 30 °C with shaking. Cultures grown overnight were diluted into fresh media to a density of OD_600_ = 0.05 and grown to OD_600_ = 0.6 before harvesting. Cells were resuspended in buffer A (50 mM Tris-Hcl, pH 7.5; 50 mM KCl; 10 mM MgCl_2_) containing 10 mM PMSF and protease inhibitor (Roche). Cells were lysed with a bead beater using glass beads and were briefly spun at 3,000 rpm for 3 min to sediment cell debris. Lysates were incubated with SDS (final SDS concentration 1%) and triton X-100 (final concentration 0.5%) and centrifuged for 1 h at 40,000 rpm. Supernatant and pellet fractions were recovered. The total cell extracts, soluble, and pellet fractions of each sample were analyzed by SDS–PAGE and immunoblot analysis using an antibody, anti-Sup35C and C-terminal specific Mcc antibody.

### Statistical Analysis

The significance of differences between the Mcc bacteriotoxic activity under different conditions was analyzed by two-way analysis of variance (ANOVA) using time and treatment as the variables. To assess differences in specific time points the data was analyzed by the Bonferroni post-test. Student’s *t-*test was used to analyze differences of activity between untreated, purified Mcc^a^ and Mcc^ia^ (Fig. 1E). The level of significance was set at P < 0.05.

## Additional Information

**How to cite this article:** Shahnawaz, M. *et al*. Prion-like characteristics of the bacterial protein MICROCIN E492. *Sci. Rep.* **7**, 45720; doi: 10.1038/srep45720 (2017).

**Publisher's note:** Springer Nature remains neutral with regard to jurisdictional claims in published maps and institutional affiliations.

## Supplementary Material

Supplementary Materials

## References

[b1] Griffith J. S. Self-replication and scrapie. Nature 215, 1043–1044 (1967). 4964084 10.1038/2151043a0

[b2] Prusiner S. B. Novel proteinaceous infectious particles cause scrapie. Science 216, 136–144 (1982). 6801762 10.1126/science.6801762

[b3] Collinge J. Prion diseases of humans and animals: their causes and molecular basis. Annu. Rev. Neurosci. 24, 519–550 (2001). 11283320 10.1146/annurev.neuro.24.1.519

[b4] Prusiner S. B. Prions. Proc. Natl. Acad. Sci. USA 95, 13363–13383 (1998). 9811807 10.1073/pnas.95.23.13363 PMC33918

[b5] Soto C. Unfolding the role of protein misfolding in neurodegenerative diseases. Nat. Rev. Neurosci. 4, 49–60 (2003). 12511861 10.1038/nrn1007

[b6] Chiti F. & Dobson C. M. Protein misfolding, functional amyloid, and human disease. Annu. Rev. Biochem. 75, 333–366 (2006). 16756495 10.1146/annurev.biochem.75.101304.123901

[b7] Soto C., Estrada L. & Castilla J. Amyloids, prions and the inherent infectious nature of misfolded protein aggregates. Trends Biochem. Sci. 31, 150–155 (2006). 16473510 10.1016/j.tibs.2006.01.002

[b8] Soto C. Transmissible proteins: expanding the prion heresy. Cell 149, 968–977 (2012). 22632966 10.1016/j.cell.2012.05.007 PMC3367461

[b9] Jarrett J. T. & Lansbury P. T. Jr. Seeding “one-dimensional crystallization” of amyloid: a pathogenic mechanism in Alzheimer’s disease and scrapie? Cell 73, 1055–1058 (1993). 8513491 10.1016/0092-8674(93)90635-4

[b10] Wickner R. B. [URE3] as an altered URE2 protein: evidence for a prion analog in Saccharomyces cerevisiae. Science 264, 566–569 (1994). 7909170 10.1126/science.7909170

[b11] Lindquist S. Mad cows meet psi-chotic yeast: the expansion of the prion hypothesis. Cell 89, 495–498 (1997). 9160741 10.1016/s0092-8674(00)80231-7

[b12] Wickner R. B., Edskes H. K., Maddelein M. L., Taylor K. L. & Moriyama H. Prions of yeast and fungi. Proteins as genetic material. J. Biol. Chem. 274, 555–558 (1999). 9872986 10.1074/jbc.274.2.555

[b13] Derkatch I. L., Bradley M. E., Hong J. Y. & Liebman S. W. Prions affect the appearance of other prions: the story of [PIN(+)]. Cell 106, 171–182 (2001). 11511345 10.1016/s0092-8674(01)00427-5

[b14] Nakayashiki T., Kurtzman C. P., Edskes H. K. & Wickner R. B. Yeast prions [URE3] and [PSI + ] are diseases. Proc. Natl. Acad. Sci. USA 102, 10575–10580 (2005). 16024723 10.1073/pnas.0504882102 PMC1180808

[b15] Eaglestone S. S., Cox B. S. & Tuite M. F. Translation termination efficiency can be regulated in Saccharomyces cerevisiae by environmental stress through a prion-mediated mechanism. EMBO J. 18, 1974–1981 (1999). 10202160 10.1093/emboj/18.7.1974 PMC1171282

[b16] True H. L. & Lindquist S. L. A yeast prion provides a mechanism for genetic variation and phenotypic diversity. Nature 407, 477–483 (2000). 11028992 10.1038/35035005

[b17] Halfmann R., Alberti S. & Lindquist S. Prions, protein homeostasis, and phenotypic diversity. Trends Cell Biol. 20, 125–133 (2010). 20071174 10.1016/j.tcb.2009.12.003 PMC2846750

[b18] Si K., Lindquist S. & Kandel E. R. A neuronal isoform of the aplysia CPEB has prion-like properties. Cell 115, 879–891 (2003). 14697205 10.1016/s0092-8674(03)01020-1

[b19] Hou F. . MAVS forms functional prion-like aggregates to activate and propagate antiviral innate immune response. Cell 146, 448–461 (2011). 21782231 10.1016/j.cell.2011.06.041 PMC3179916

[b20] de Lorenzo V. Isolation and characterization of microcin E492 from Klebsiella pneumoniae. Arch. Microbiol. 139, 72–75 (1984). 6385903 10.1007/BF00692715

[b21] de Lorenzo V., Martinez J. L. & Asensio C. Microcin-mediated interactions between Klebsiella pneumoniae and Escherichia coli strains. J. Gen. Microbiol. 130 (Pt 2), 391–400 (1984). 6374023 10.1099/00221287-130-2-391

[b22] Destoumieux-Garzon D. . Microcin E492 antibacterial activity: evidence for a TonB-dependent inner membrane permeabilization on Escherichia coli. Mol. Microbiol. 49, 1031–1041 (2003). 12890026 10.1046/j.1365-2958.2003.03610.x

[b23] de Lorenzo V. & Pugsley A. P. Microcin E492, a low-molecular-weight peptide antibiotic which causes depolarization of the Escherichia coli cytoplasmic membrane. Antimicrob. Agents Chemother. 27, 666–669 (1985). 2408563 10.1128/aac.27.4.666 PMC180121

[b24] Lagos R., Wilkens M., Vergara C., Cecchi X. & Monasterio O. Microcin E492 forms ion channels in phospholipid bilayer membrane. FEBS Lett. 321, 145–148 (1993). 7682973 10.1016/0014-5793(93)80096-d

[b25] de Lorenzo V. Factors affecting microcin E492 production. J. Antibiot. 38, 340–345 (1985). 3924870 10.7164/antibiotics.38.340

[b26] Corsini G., Baeza M., Monasterio O. & Lagos R. The expression of genes involved in microcin maturation regulates the production of active microcin E492. Biochimie 84, 539–544 (2002). 12423798 10.1016/s0300-9084(02)01415-3

[b27] Bieler S. . Amyloid formation modulates the biological activity of a bacterial protein. J. Biol. Chem. 280, 26880–26885 (2005). 15917245 10.1074/jbc.M502031200

[b28] Ghetti B. . Prion protein amyloidosis. Brain Pathol. 6, 127–145 (1996). 8737929 10.1111/j.1750-3639.1996.tb00796.x

[b29] Yang H., Yang S., Kong J., Dong A. & Yu S. Obtaining information about protein secondary structures in aqueous solution using Fourier transform IR spectroscopy. Nat. Protoc. 10, 382–396 (2015). 25654756 10.1038/nprot.2015.024

[b30] Kayed R. . Common structure of soluble amyloid oligomers implies common mechanism of pathogenesis. Science 300, 486–489 (2003). 12702875 10.1126/science.1079469

[b31] Lorenzo A. & Yankner B. A. Beta-amyloid neurotoxicity requires fibril formation and is inhibited by congo red. Proc. Natl. Acad. Sci. USA 91, 12243–12247 (1994). 7991613 10.1073/pnas.91.25.12243 PMC45413

[b32] Porat Y., Abramowitz A. & Gazit E. Inhibition of amyloid fibril formation by polyphenols: structural similarity and aromatic interactions as a common inhibition mechanism. Chem. Biol. Drug Des 67, 27–37 (2006). 16492146 10.1111/j.1747-0285.2005.00318.x

[b33] Yang F. . Curcumin inhibits formation of amyloid beta oligomers and fibrils, binds plaques, and reduces amyloid *in vivo*. J. Biol. Chem. 280, 5892–5901 (2005). 15590663 10.1074/jbc.M404751200

[b34] Caughey B., Raymond G. J., Kocisko D. A. & Lansbury P. T. Jr. Scrapie infectivity correlates with converting activity, protease resistance, and aggregation of scrapie-associated prion protein in guanidine denaturation studies. J. Virol. 71, 4107–4110 (1997). 9094691 10.1128/jvi.71.5.4107-4110.1997 PMC191566

[b35] Li L. & Lindquist S. Creating a protein-based element of inheritance. Science 287, 661–664 (2000). 10650001 10.1126/science.287.5453.661

[b36] Osherovich L. Z., Cox B. S., Tuite M. F. & Weissman J. S. Dissection and design of yeast prions. PLoS. Biol. 2, E86 (2004). 15045026 10.1371/journal.pbio.0020086 PMC374241

[b37] Chernoff Y. O., Lindquist S. L., Ono B., Inge-Vechtomov S. G. & Liebman S. W. Role of the chaperone protein Hsp104 in propagation of the yeast prion-like factor [psi+]. Science 268, 880–884 (1995). 7754373 10.1126/science.7754373

[b38] Ferreira P. C., Ness F., Edwards S. R., Cox B. S. & Tuite M. F. The elimination of the yeast [PSI+] prion by guanidine hydrochloride is the result of Hsp104 inactivation. Mol. Microbiol. 40, 1357–1369 (2001). 11442834 10.1046/j.1365-2958.2001.02478.x

[b39] Jung G. & Masison D. C. Guanidine hydrochloride inhibits Hsp104 activity *in vivo*: a possible explanation for its effect in curing yeast prions. Curr. Microbiol. 43, 7–10 (2001). 11375656 10.1007/s002840010251

[b40] Patino M. M., Liu J. J., Glover J. R. & Lindquist S. Support for the prion hypothesis for inheritance of a phenotypic trait in yeast. Science 273, 622–626 (1996). 8662547 10.1126/science.273.5275.622

[b41] Tagliavini F., Forloni G., D’Ursi P., Bugiani O. & Salmona M. Studies on peptide fragments of prion proteins. Adv. Protein Chem. 57, 171–201 (2001). 11447690 10.1016/s0065-3233(01)57022-9

[b42] Holscher C., Delius H. & Burkle A. Overexpression of nonconvertible PrPc delta114-121 in scrapie-infected mouse neuroblastoma cells leads to trans-dominant inhibition of wild-type PrP(Sc) accumulation. J. Virol. 72, 1153–1159 (1998). 9445012 10.1128/jvi.72.2.1153-1159.1998 PMC124590

[b43] Caughey B. & Lansbury P. T. Protofibrils, pores, fibrils, and neurodegeneration: separating the responsible protein aggregates from the innocent bystanders. Annu. Rev. Neurosci. 26, 267–298 (2003). 12704221 10.1146/annurev.neuro.26.010302.081142

[b44] Kane M. D. . Evidence for seeding of beta -amyloid by intracerebral infusion of Alzheimer brain extracts in beta -amyloid precursor protein-transgenic mice. J. Neurosci. 20, 3606–3611 (2000). 10804202 10.1523/JNEUROSCI.20-10-03606.2000 PMC6772682

[b45] Meyer-Luehmann M. . Exogenous induction of cerebral beta-amyloidogenesis is governed by agent and host. Science 313, 1781–1784 (2006). 16990547 10.1126/science.1131864

[b46] Morales R., Duran-Aniotz C., Castilla J., Estrada L. D. & Soto C. De novo induction of amyloid-beta deposition *in vivo*. Mol. Psychiatry 17, 1347–1353 (2012). 21968933 10.1038/mp.2011.120

[b47] Rymer D. L. & Good T. A. The role of prion peptide structure and aggregation in toxicity and membrane binding. J. Neurochem. 75, 2536–2545 (2000). 11080207 10.1046/j.1471-4159.2000.0752536.x

[b48] Ettaiche M., Pichot R., Vincent J. P. & Chabry J. *In vivo* cytotoxicity of the prion protein fragment 106-126. J. Biol. Chem. 275, 36487–36490 (2000). 11007766 10.1074/jbc.C000579200

[b49] Brown D. R. PrPSc-like prion protein peptide inhibits the function of cellular prion protein. Biochem. J. 352 Pt 2, 511–518 (2000). 11085945 PMC1221483

[b50] Chabry J., Caughey B. & Chesebro B. Specific inhibition of *in vitro* formation of protease-resistant prion protein by synthetic peptides. J. Biol. Chem. 273, 13203–13207 (1998). 9582363 10.1074/jbc.273.21.13203

[b51] Soto C. . Reversion of prion protein conformational changes by synthetic beta-sheet breaker peptides. Lancet 355, 192–197 (2000). 10675119 10.1016/s0140-6736(99)11419-3

[b52] Shahnawaz M. & Soto C. Microcin amyloid fibrils A are reservoir of toxic oligomeric species. J. Biol. Chem. 287, 11665–11676 (2012). 22337880 10.1074/jbc.M111.282533 PMC3320916

[b53] Wray W., Boulikas T., Wray V. P. & Hancock R. Silver staining of proteins in polyacrylamide gels. Anal. Biochem. 118, 197–203 (1981). 6175245 10.1016/0003-2697(81)90179-2

[b54] LeVine H. III Thioflavine T interaction with synthetic Alzheimer’s disease beta-amyloid peptides: detection of amyloid aggregation in solution. Protein Sci. 2, 404–410 (1993). 8453378 10.1002/pro.5560020312 PMC2142377

[b55] Mayr-Harting A., Hedges A. J. & Berkeley R. C. W. Methods for studying bacteriocins. Methods Microbiol. 7A, 315–422 (1972).

