# Supplementary material for: Prion-like characteristics of the bacterial protein Microcin E492
Source: Sci Rep. 2017 Mar 31;7:45720. doi: 10.1038/srep45720 (PMC5374632; doi:10.1038/srep45720)

SUPPLEMENTARY INFORMATION

PRION-LIKE CHARACTERISTICS OF THE BACTERIAL PROTEIN MICROCIN E492

Mohammad Shahnawaz, Kyung-Won Park, Abhisek Mukherjee,

Rodrigo Diaz-Espinoza, and Claudio Soto

SUPPLEMENTARY FIGURE LEGENDS:

__Figure S1.__ **Immunodepletion of Mcc^{ia} from culture medium.** Aliquots of culture medium before (*lane* 1) and after immunodepletion (*lane 2*) were resolved by NuPAGE 4-12% Bis-Tris gel. Proteins were electrophoretically transferred to nitrocellulose membrane. The membrane was probed with anti-Mcc antibody and the anti-rabbit secondary antibodies. The blot was visualized using enhanced chemiluminescence plus western blotting detection kit. The numbers on the left indicate the relative molecular weights of protein markers in kilo Dalton (kDa).

__Figure S2__. **The time in which Mcc^{ia} was added to the culture affects conversion of Mcc^{a} to Mcc^{ia} *in vivo*. A**: Schematic of Mcc^{ia} addition time during the growth of Mcc producing bacteria. **B**: Mcc producing bacteria were grown in minimal medium at 37 °C until 48 h either in the absence (*control*), or in the presence of 20% (v/v) culture supernatant containing Mcc^{ia} added at different time points (0, 3 and 6 h). Aliquots were removed at the indicated times and activity of Mcc was measured by the critical dilution method. *Error bars* indicate S.D. The differences in Mcc activity between control and treated samples were highly significant (P < 0.0001) in both variables (time and treatment, as well as the interaction between them) as measured by two-way ANOVA. Post-hoc analysis by the Bonferroni test revealed also significant differences between treatment and controls at times 9 and 12 h (***P < 0.001).

__Figure S3__. **Mcc^{ia} propagates *in vivo* after repeated passaging**. **A**: Schematic representation of the procedure for Mcc^{ia} passaging *in vivo*. **B**: Mcc producing bacteria were grown in minimal medium at 37 °C until 48 h either in the absence (*control*), or in the presence of 10% (v/v) wholeculture containing Mcc^{ia} (*passage* 1), and then after every 48 h this cycle was repeated until (*passage* 2 and 3). In each cycle, aliquots were removed at the indicated times and activity of Mcc was measured by the critical dilution method. In each case, samples were used in duplicates. *Error bars* indicate S.D. The differences in Mcc activity between control and treated samples were analyzed by two-way ANOVA followed by Bonferroni post-test (*** P < 0.001).

__Figure S4__. **Schematic representation of Mcc^{ia} preparation and seeding *in vivo***. **A**: To prepare Mcc^{ia} from the culture of Mcc producing bacteria, a 1:1000 dilution of overnight culture of Mcc producing bacteria was inoculated in M9 minimal medium and grown for 48 h (stationary phase) at 37 °C with shaking. After 48 h, either whole bacterial culture (W) was used as such for

seeding or culture supernatant (S) after removing bacterial cells by centrifuging at 4000 rpm for 10 minutes. **B**: For *in vivo* seeding, a 1:1000 dilution of overnight culture of Mcc producing bacteria was inoculated in M9 minimal medium in presence or absence of purified Mcc^{ia} or culture containing Mcc^{ia}, and grown for 48 h at 37 °C with shaking. Aliquots were removed periodically and used for Mcc activity assay by the critical dilution method and for limited proteolysis by proteinase K.

Supplementary Figure 1

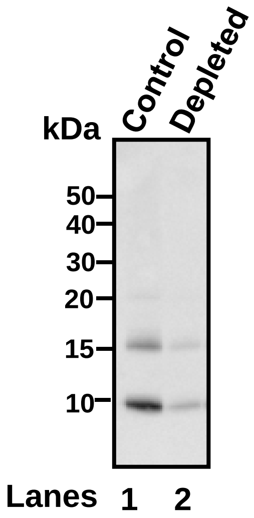

Supplementary Figure 2

A

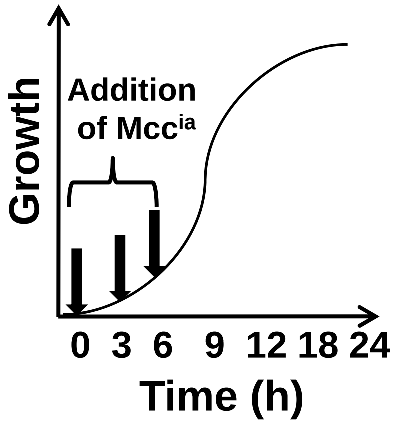

B

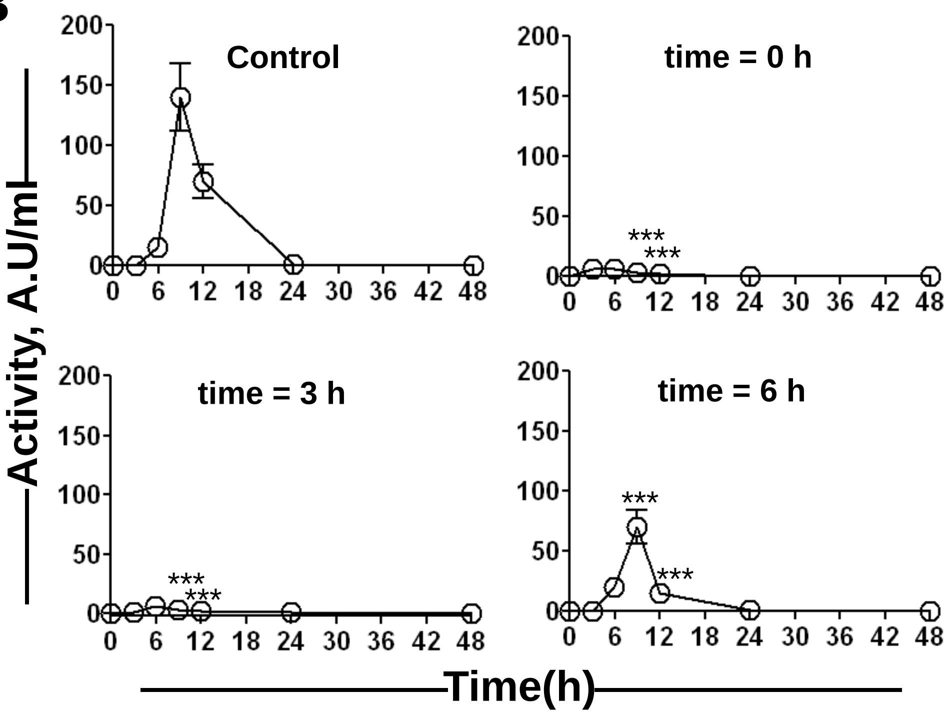

Supplementary Figure 3

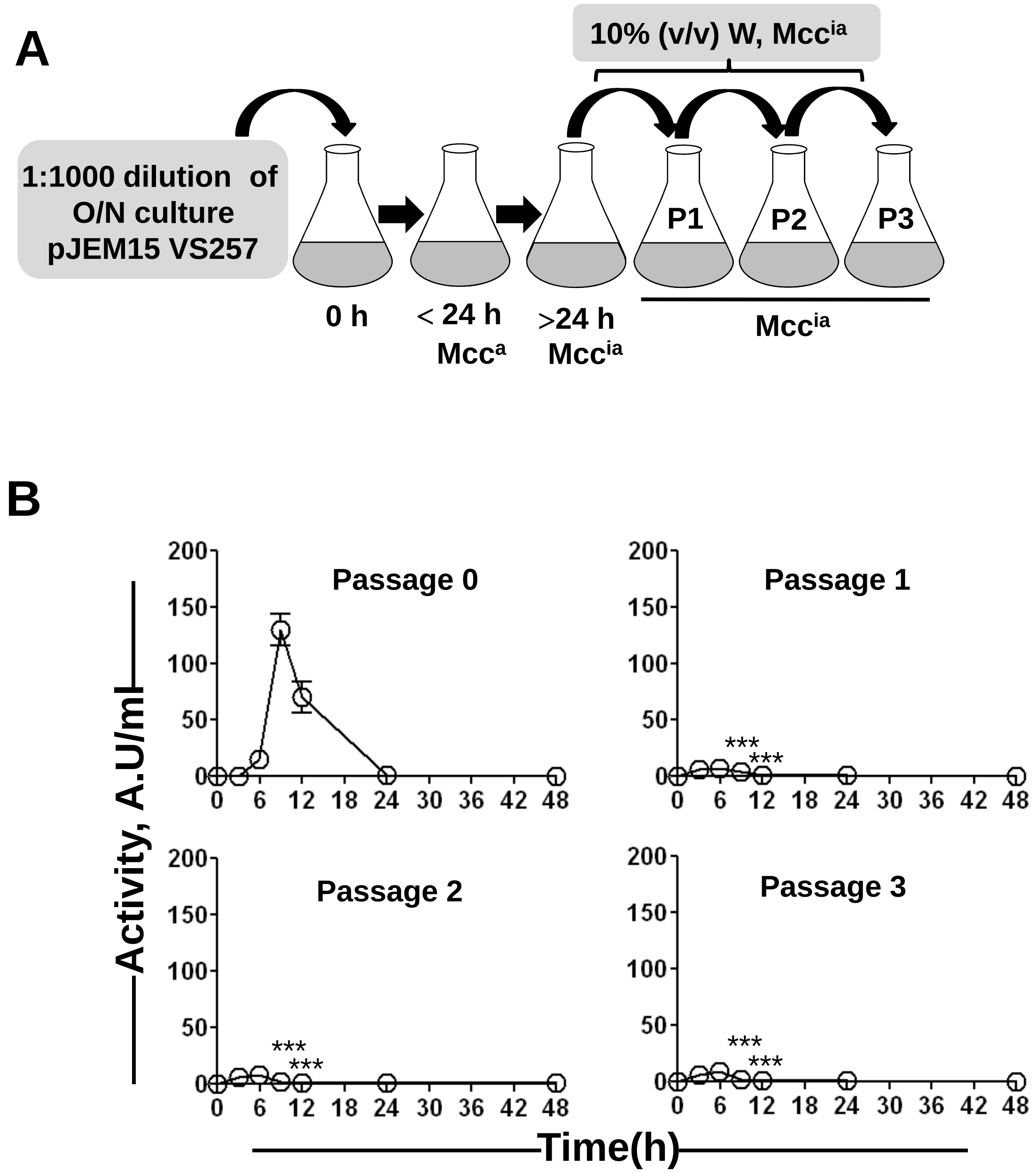

Supplementary Figure 4

A **Preparation of Mcc^{ia} from bacterial cultures:**

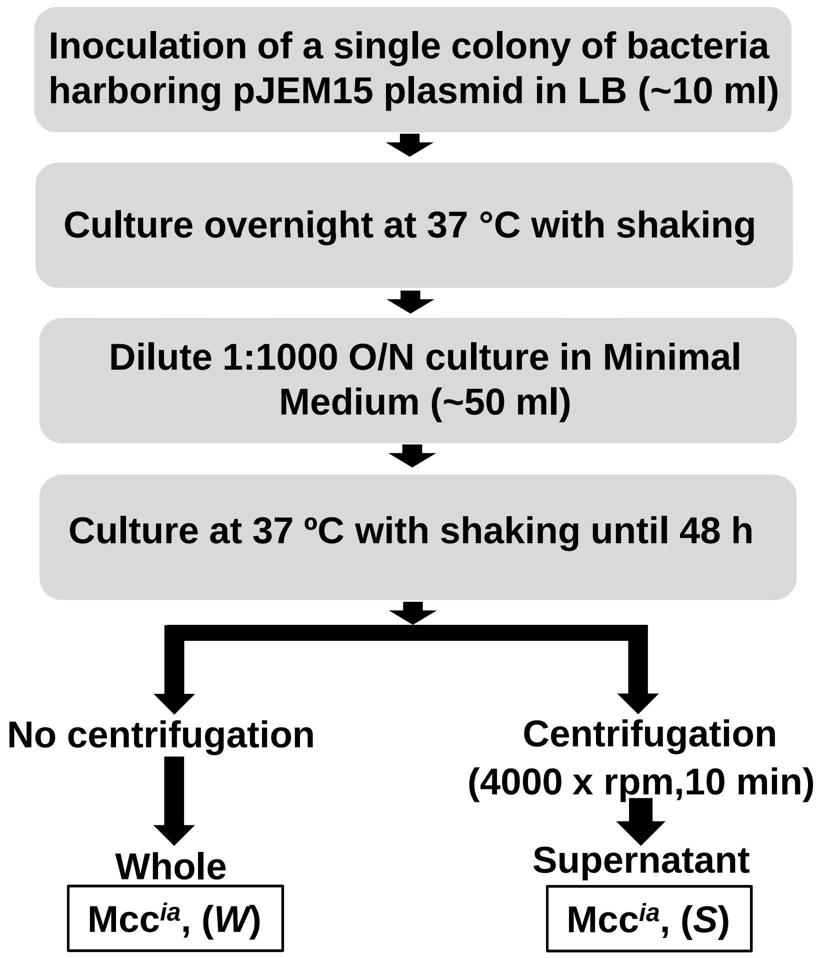

B **Seeding *in vivo:***

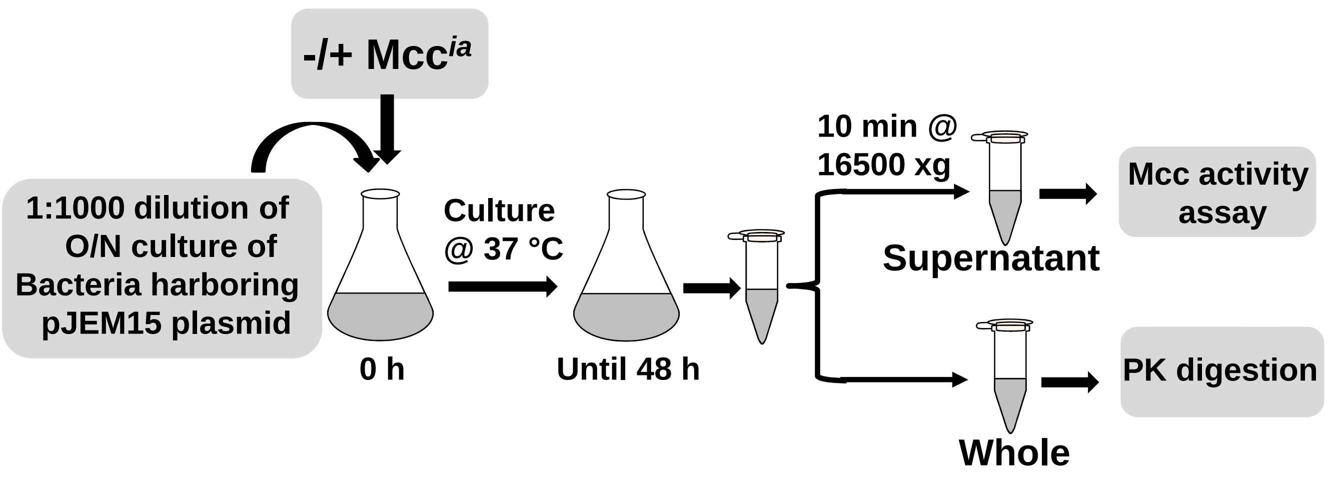

Supplement: Supplementary Materials [file srep45720-s1.pdf]
